# Robot-Assisted Radical Prostatectomy After Rezūm: A Case Report and Literature Review

**DOI:** 10.3390/life16020362

**Published:** 2026-02-21

**Authors:** Kosta Cerović, Simon Hawlina

**Affiliations:** 1 Department of Urology, University Medical Centre Ljubljana, 1000 Ljubljana, Slovenia; kosta.cerovic@kclj.si; 2 Department of Surgery, Faculty of Medicine, University of Ljubljana, 1000 Ljubljana, Slovenia

**Keywords:** prostate cancer, robotic surgery, robot-assisted radical prostatectomy (RARP), Rezūm, benign prostatic obstruction (BPO), intraoperative fibrosis, minimally invasive surgical therapies (MISTs)

## Abstract

Minimally invasive surgical therapies (MISTs), such as Rezūm™ Water Vapor Therapy, are emerging treatment options for benign prostatic obstruction (BPO). When prostate cancer is subsequently diagnosed, radical prostatectomy may still be indicated. However, evidence regarding intraoperative challenges and the surgical and functional outcomes of robot-assisted radical prostatectomy (RARP) following Rezūm remains limited. We report the first documented case of RARP following Rezūm in a 68-year-old man. He initially underwent Rezūm for symptomatic BPO. Due to rising PSA, a suspicious lesion on MRI, and a biopsy-confirmed high-risk prostate carcinoma, radical surgery was performed. Intraoperatively, dense fibrosis and altered tissue planes required precise dissection and a level 2 bilateral nerve-sparing approach. A systematic review revealed no previously published cases of RARP after Rezūm. On the other hand, RARP after transurethral resection of the prostate (TURP) is associated with increased operative time, blood loss, and bladder neck reconstruction, though late continence and biochemical recurrence rates are similar to those in treatment-naïve patients. In conclusion, RARP after ablative BPO therapies is feasible but may present unique technical challenges. Larger prospective studies are needed to develop standardized management strategies.

## 1. Introduction

Benign prostatic obstruction (BPO) affects up to 50% of men by age 60 and frequently causes lower urinary tract symptoms (LUTSs). Rezūm™, a water vapor-based therapy, provides a minimally invasive outpatient treatment option that reduces International Prostate Symptom Score (IPSS) by roughly 50% or more and improves peak urinary flow by 4–5 mL/s, with these benefits remaining durable over at least five years and retreatment rates staying low at about 4–5% [1]. The procedure has a favorable safety profile in which most adverse events are mild or transient, while serious complications such as transfusion-requiring bleeding or persistent sexual dysfunction are rare [1,2,3]. Importantly, Rezūm preserves both erectile and ejaculatory function in most patients, setting it apart from traditional surgical options that carry higher risks of sexual side effects [1].

Patients who are suspected of having or diagnosed with prostate cancer should not undergo Rezūm therapy without comprehensive assessment, since this minimally invasive procedure does not yield tissue for pathological analysis and may overlook the presence of malignancy while treating BPO. However, with increasing use of Rezūm, clinicians may encounter prostate cancer in patients previously treated with this modality. Even accurate detection and characterization of residual or recurrent prostate cancer in this setting remain challenging. Though PSA testing is vital in prostate cancer care, its specificity for malignancy versus ablation-related benign changes is limited; this reinforces the importance of interpreting PSA levels together with comprehensive clinical evaluation and histological confirmation [4].

Robot-assisted radical prostatectomy (RARP) remains the standard of care for localized prostate cancer. Prior prostatic surgeries, especially transurethral resection of the prostate (TURP), are known to induce periprostatic fibrosis, leading to prolonged operative time, increased blood loss, and potential need for bladder neck reconstruction, although long-term oncologic and continence outcomes remain comparable to TURP-naïve patients [5]. To date, no case reports have described RARP following Rezūm. Here, we present our initial experience and review relevant studies in the literature on RARP after minimally invasive surgical therapies (MISTs).

## 2. Case Presentation

A 68-year-old man with a history of left total hip arthroplasty presented in February 2023 with LUTS (IPSS 11; storage subscore 2), peak urinary flow of 12 mL/s (voided volume: 150 mL), post-void residual of ~20 mL, and PSA of 3 ng/mL. Digital rectal examination revealed a firm nodule in the left prostate base. Multiparametric MRI (21 February 2023) showed a 61 mL prostate (PSAD 0.05) with a left paramedian lesion. Diffusion-weighted imaging was non-diagnostic due to hip prosthesis artifacts. He underwent Rezūm (Boston Scientific Company Inc., Marlborough, MA, USA) therapy on 30 May 2023, which relieved urgency, though voided volumes remained at ~200 mL.

By 29 February 2024, PSA had increased to 7 ng/mL and subsequently to 16.8 ng/mL by 4 December 2024. Repeat MRI (24 December 2024) showed a 50 mL prostate and a persistent left paramedian lesion, though DWI remained non-diagnostic. A positive SelectMDx test prompted transperineal biopsy on 9 January 2025, which confirmed Gleason 9 (5 + 4) adenocarcinoma in 8 of 10 cores. Choline PET-CT suggested left seminal vesicle invasion and iliac nodal uptake, with no evidence of bone metastases.

The patient received 150 mg of bicalutamide daily at the referring institution prior to referral to our center, where he underwent RARP using the da Vinci Xi Surgical System (Intuitive Surgical Inc., Sunnyvale, CA, USA) on 17 April 2025. We acknowledge that neoadjuvant antiandrogen monotherapy does not constitute guideline-recommended standard treatment for high-risk localized prostate cancer. Dense fibrosis and distorted tissue planes were encountered intraoperatively. Given the history of prior Rezūm therapy and the presence of locally advanced disease, the origin of these fibrotic changes was likely multifactorial. A level 2 (interfascial), bilateral nerve-sparing approach was completed successfully. Although bladder neck reconstruction was anticipated, it was not required. Estimated blood loss was within expected limits, and there were no intraoperative complications. A cystogram on postoperative day 12 (29 April 2025) showed a watertight vesicourethral anastomosis; the catheter was removed the same day.

**Figure 1:**
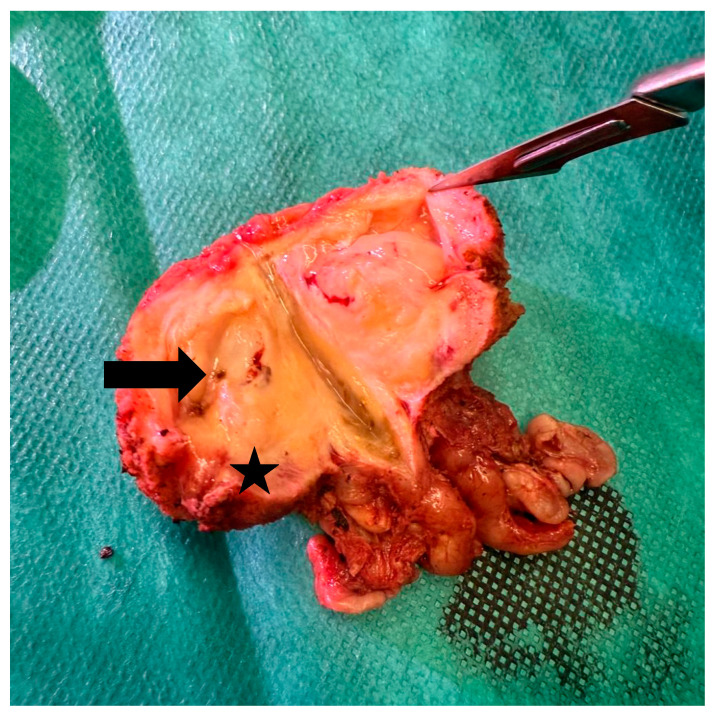
Prostatectomy specimen after RARP. The arrow indicates post-Rezūm pale fibrotic changes within the peripheral zone. The star (*) denotes the suspected area of carcinoma (R1 resection margin).

Final pathology revealed prostate adenocarcinoma, Gleason 7 (4 + 3), with prominent cribriform architecture and a tertiary Gleason 5 component (Figure 1). The tumor was multifocal, involving over 30% of the prostatic volume, with lymphovascular invasion, perineural invasion, extracapsular extension, and bilateral seminal vesicle involvement. A positive surgical margin (8 mm) was noted at the right anterior apex (Gleason 4). One left pelvic lymph node was positive. Final stage: pT3b N1 R1.

We observed postoperative PSA persistence of 0.144 ng/mL, which rose to 0.410 ng/mL by 20 August 2025. A PSMA PET-CT performed on 17 October 2025 demonstrated signs of prostate carcinoma spread with increased PSMA expression in regional lymph nodes and in a lymph node at the bifurcation of the right common iliac vessels. The patient started LHRH antagonist therapy with relugolix and is awaiting discussion at the uro-oncological multidisciplinary team meeting for therapy intensification.

Functional outcomes at 3 months after surgery showed complete continence (no PADS used), and the patient reported complete erectile dysfunction.

## 3. Literature Review

A PubMed search with the query ((Rezūm) OR (Rezum) OR (Water Vapor Thermal Therapy) OR (WVTT)) AND (radical prostatectomy) revealed no published accounts of radical prostatectomy performed following Rezūm therapy. This underscores the novelty of the reported case, as published evidence is absent for the specific scenario of RARP after Rezūm.

By contrast, the impact of previous transurethral resection of the prostate (TURP) on subsequent RARP has been rigorously examined. Veccia et al. [6] performed a meta-analysis encompassing 12 studies and found that prior TURP or BPO procedures were associated with longer operative times, increased blood loss, and greater need for bladder neck reconstruction, as well as prolonged catheterization. Despite these intraoperative challenges, key outcomes such as 12-month continence rates (76–82%) and biochemical recurrence were not significantly affected [6]. Carbin et al. [7] matched patients undergoing RARP after TURP with controls and noted no distinct differences in positive surgical margin rates, biochemical recurrence, or postoperative incontinence, suggesting comparable oncologic and functional outcomes. Gu et al. [8] corroborated these findings, showing that a history of TURP did not adversely impact long-term oncologic or functional RARP outcomes.

Collectively, these studies indicate that while prior surgical interventions can complicate RARP technique and increase perioperative risks, the long-term oncologic and functional results generally remain favorable when performed at high-volume centers by experienced surgeons.

## 4. Discussion

To the best of our knowledge, this paper represents the first documented case of RARP performed following Rezūm water vapor therapy, marking an important contribution to the limited literature on radical prostatectomy after MISTs for BPO.

Data regarding RARP after other MISTs are also scarce. Notably, Moschovas et al. [9] described RARP after UroLift^®^, highlighting technical complexities such as the presence of metal UroLift^®^ clips, chronic inflammation, and distortion of anatomical planes, which can pose intraoperative challenges. These factors mandate careful clip removal and avoidance of electrocautery near foreign bodies to reduce the risk of thermal injury [9]. With respect to Aquablation, the literature currently lacks reports of RARP in patients previously treated with this modality. However, studies suggest that Aquablation remains effective for recurrent symptoms post-UroLift^®^ and may be safely performed as a secondary intervention [10]. There is a clear gap in published data for RARP following Rezūm and Aquablation specifically, which further emphasizes the significance of this case report and review of literature.

It is important to clarify that the initial Rezūm procedure and pre-interventional management were performed at an external institution. Our center became involved only after the diagnosis of prostate cancer had been established and the patient was referred for surgical treatment. Nevertheless, this case underscores a crucial oncologic principle: suspicious findings on digital rectal examination or multiparametric MRI warrant histologic confirmation prior to any minimally invasive therapy for BPO, regardless of PSA level. In retrospect, the absence of a biopsy before Rezūm therapy may have contributed to a delayed diagnosis of high-risk prostate cancer in this patient. This case therefore reinforces the necessity of strict oncologic evaluation before performing MIST procedures.

Clear extrapolation from ablative therapies or TURP to Rezūm-treated patients cannot be safely performed as they are completely different techniques. However, broader literature on RARP after prior benign prostate procedures, such as TURP and holmium laser enucleation (HoLEP), further contextualizes potential operative challenges. Recent meta-analyses show that preceding interventions like TURP are associated with increased technical difficulty: operative time, blood loss, hospitalization, need for bladder neck reconstruction, and major complications are all elevated, while nerve-sparing success, continence recovery, and early potency rates are reduced [11]. The positive surgical margin rate is also moderately higher in the post-TURP group [11]. Importantly, long-term cancer control and rates of transfusion, lymphadenectomy, minor complications, unilateral nerve-sparing, and biochemical recurrence remain comparable to those of standard RARP procedures [11]. Patients undergoing RP after HoLEP similarly demonstrate higher intraoperative complication rates, more frequent urethrovesical anastomosis leaks, and lower urinary continence after surgery than controls, although erectile function and pathological outcomes are not significantly compromised [12].

Importantly, different studies show that nearly every tenth patient undergoing surgery for LUTS without previous prostate biopsy harbors incidental prostate cancer [13,14]. Consequently, one can assume that there are (or there will be) numerous cases of prostate cancer following new ablative and minimally invasive therapies, but it is most likely that the occurrence is currently underreported.

Rezūm steam ablation induces targeted tissue necrosis predominantly within the transition zone, as it is designed to treat transitional and central prostatic adenomas, followed by inflammatory remodeling and fibrosis [3,15,16,17]. In the present case, marked fibrosis was observed in the peripheral zone and periprostatic tissues. Given the final pathological stage (pT3b with seminal vesicle invasion), these findings cannot be attributed exclusively to prior Rezūm therapy. Tumor-associated desmoplastic stromal reaction in locally advanced prostate cancer likely contributed to distortion of tissue planes. Accordingly, the intraoperative fibrosis should be regarded as multifactorial in origin.

The transition zone and prostatic urethra were not significantly altered or widened, which may account for the limited symptomatic response after Rezūm therapy. The zonal distribution of fibrosis had relevant intraoperative implications, as fibrotic changes in the peripheral region and around the prostatic capsule required meticulous dissection and careful preservation of neurovascular structures. Nevertheless, level 2 (interfascial) bilateral nerve-sparing RARP was completed without intraoperative complications, bladder neck reconstruction, or excessive blood loss. This case underscores the importance of detailed preoperative imaging and careful intraoperative assessment when radical prostatectomy is performed after prior ablative therapy. Postoperative catheterization was prolonged; however, cystography on postoperative day 12 confirmed a watertight vesicourethral anastomosis.

Based on our experience with RARP after Rezūm therapy, we hypothesize that intraoperative complexity may be influenced by factors such as the number of steam injections or treated sites, the anatomical position and spacing of these injections, and the time interval between ablation and prostatectomy. While definitive evidence is lacking, it seems plausible that patients who have undergone more extensive or multiple treatments could develop broader areas of fibrosis, potentially leading to obscured dissection planes, complicated anatomical orientation during surgery and, in some cases, requiring customized dissection and reconstructive strategies, prolonging the duration of the surgery. Further studies are needed to clarify the impact of these variables on surgical outcomes.

The temporal relationship between Rezūm and RARP can also play an important role. Surgery conducted soon after TURP (e.g., within three to six months) is confronted with ongoing inflammatory changes and residual edema, which increase tissue friability and the risk of intra- and postoperative complications [18,19]. Conversely, operations delayed twelve months or longer typically encounter mature, fibrotic tissue, which, while less susceptible to bleeding, demands sharp dissection and may restrict mobility during anatomical reconstruction, particularly at the bladder neck and apex [20,21]. Our perioperative approach was adapted according to these variables, underscoring the necessity for thorough preoperative review of prior Rezūm therapy records and detailed imaging assessment.

Limitations of this study include its nature as a single case report, as well as short-term follow-up. More data are needed regarding long-term continence, erectile function, and oncologic control after RARP in post-Rezūm patients. Prospectively, multicenter trials and registries are needed to refine surgical strategies and inform evidence-based patient counseling for this emerging, increasingly relevant cohort. For clinicians, these findings underscore the necessity of careful preoperative workup, patient-centered counseling about possible perioperative and functional risks, and ongoing surveillance.

Looking ahead, the continuous evolution of surgical technologies—including single-port robotics, AI-assisted approaches, and organ-sparing techniques—is already shaping global guidelines and driving a shift toward more individualized, patient-centered, and minimally invasive prostate cancer management [22]. Transurethral ablation therapies like Rezūm may further broaden therapeutic options for focal, repeatable interventions even in intermediate-risk and recurrent cancers, contingent on increased long-term evidence and refinement of patient selection criteria [23].

## 5. Conclusions

RARP following Rezūm appears technically feasible; however, surgeons should be aware of the possibility of altered tissue planes and fibrotic changes. Given the limited evidence available, individualized preoperative planning and cautious intraoperative dissection are advisable. Prospective studies with larger patient cohorts are essential to establish best practices and long-term outcomes for this increasingly relevant clinical scenario.

## 6. Take-Home Points

- After UroLift^®^: Plan for clip removal, avoid energy near clips, and anticipate altered bladder neck anatomy.
- After Rezūm™: Altered tissue planes and fibrosis may be encountered. The extent and distribution of fibrosis can be variable.
- After Aquablation: No published data currently exist on RARP following Aquablation.

## Data Availability

The raw data supporting the conclusions of this article will be made available by the authors upon request.
